# Supplementary material for: Association between unemployment and the co-occurrence and clustering of common risky health behaviors: Findings from the Constances cohort
Source: PLoS One. 2020 May 6;15(5):e0232262. doi: 10.1371/journal.pone.0232262 (PMC7202648; doi:10.1371/journal.pone.0232262)
Supplement: S1 Table — (DOCX) [file pone.0232262.s001.docx]

Table S1: Baseline sociodemographic characteristics of CONSTANCES participants compared to randomly selected workers.

|  | **CONSTANCES**  **participants** | **Randomly**  **selected individuals** | **p** |
| --- | --- | --- | --- |
| **N** | 65,630 | 14,597 | - |
| **Age, y (mean ± SD)** | 42.7 ± 10.4 | 39.9 ± 10.7 | <0.0001 |
| **Men,** **% (n)** | 48.6 (31,915) | 50.9 (7436) | <0.0001 |
| **Unemployment rate, % (n)** | 7.0 (4573) | 10.4 (1524) | <0.0001 |
| **Education, % (n)** |  |  |  |
| Primary, lower secondary | 17.6 (11,558) | 52.2 (7626) |  |
| High school diploma | 15.3 (10,034) | 16.2 (2363) | <0.0001 |
| University | 67.1 (44,038) | 31.6 (4608) |  |

Univariate comparisons were performed with chi-square test (or Fisher’s exact test when necessary) for categorical variables and with Student’s t-test for continuous variables. Randomly selected workers in the same age range (18–65 years) than CONSTANCES participants at baseline are from the decennial health survey conducted in 2003 by the French National Institute of Statistics and Economic Studies.
