# Supplementary material for: Association between unemployment and the co-occurrence and clustering of common risky health behaviors: Findings from the Constances cohort
Source: PLoS One. 2020 May 6;15(5):e0232262. doi: 10.1371/journal.pone.0232262 (PMC7202648; doi:10.1371/journal.pone.0232262)
Supplement: S2 Table — (DOCX) [file pone.0232262.s002.docx]

Table S2: Relative risk ratios of co-occurring risk factors according to experience of unemployment in Constances cohort: stratification by age, education and self-rated health, multinomial regression (reference: 0 risk).

|  |  | **1 risk** |  |  | **2 risks** |  |  | **3 risks** |  |  | **4 risks** |  |
| --- | --- | --- | --- | --- | --- | --- | --- | --- | --- | --- | --- | --- |
|  | **RRR** | **95% CI** | **p** | **RRR** | **95% CI** | **p** | **RRR** | **95% CI** | **p** | **RRR** | **95% CI** | **p** |
| **Age (reference 18-36 y.)** | | | | | | | | | | | | |
| **Never # Age 18-36** | 1 | 1.00 - 1.00 | . | 1.00 | 1.00 - 1.00 | . | 1.00 | 1.00 - 1.00 | . | 1.00 | 1.00 - 1.00 | . |
| **Past only # Age 37-47** | 0.94 | 0.79 - 1.12 | 0.47 | 0.95 | 0.80 - 1.14 | 0.60 | 0.87 | 0.70 - 1.07 | 0.19 | 0.62 | 0.41 - 0.93 | **0.022** |
| **Past only # Age 48-65** | 0.87 | 0.71 - 1.08 | 0.21 | 0.81 | 0.65 - 1.01 | 0.066 | 0.74 | 0.56 - 1.00 | **0.047** | 0.33 | 0.16 - 0.70 | **0.004** |
| **Now only # Age 37-47** | 1.3 | 0.95 - 1.80 | 0.11 | 1.29 | 0.93 - 1.80 | 0.12 | 1.63 | 1.13 - 2.36 | **0.01** | 1.91 | 1.05 - 3.46 | **0.033** |
| **Now only # Age 48-65** | 0.99 | 0.70 - 1.40 | 0.96 | 0.83 | 0.58 - 1.20 | 0.33 | 0.99 | 0.63 - 1.55 | 0.96 | 0.41 | 0.12 - 1.42 | 0.16 |
| **Past & now # Age 37-47** | 1.04 | 0.66 - 1.65 | 0.86 | 1.16 | 0.74 - 1.83 | 0.52 | 1.12 | 0.66 - 1.88 | 0.68 | 0.8 | 0.34 - 1.89 | 0.61 |
| **Past & now # Age 48-65** | 0.95 | 0.58 - 1.54 | 0.83 | 0.63 | 0.38 - 1.06 | 0.08 | 0.72 | 0.38 - 1.34 | 0.30 | 1.04 | 0.39 - 2.80 | 0.93 |
| **Education (Reference: Primary)** | | | | | | | | | | | | |
| **Never # Primary** | 1 | 1.00 - 1.00 | . | 1 | 1.00 - 1.00 | . | 1 | 1.00 - 1.00 | . | 1 | 1.00 - 1.00 | . |
| **Past only # Secondary** | 1.01 | 0.78 - 1.30 | 0.94 | 1.05 | 0.81 - 1.37 | 0.69 | 1 | 0.73 - 1.36 | 0.99 | 1.47 | 0.83 - 2.60 | 0.19 |
| **Past only # University** | 1.25 | 1.03 - 1.52 | **0.03** | 1.28 | 1.05 - 1.57 | **0.02** | 1.38 | 1.09 - 1.76 | **0.01** | 1.51 | 0.95 - 2.39 | 0.082 |
| **Now only # Secondary** | 1.24 | 0.78 - 1.98 | 0.37 | 1.08 | 0.67 - 1.72 | 0.75 | 1.17 | 0.70 - 1.94 | 0.55 | 0.87 | 0.37 - 2.03 | 0.75 |
| **Now only # University** | 1.31 | 0.92 - 1.88 | 0.14 | 1.04 | 0.73 - 1.49 | 0.82 | 1.01 | 0.68 - 1.50 | 0.96 | 1.02 | 0.55 - 1.91 | 0.95 |
| **Past & now # Secondary** | 0.65 | 0.35 - 1.21 | 0.17 | 0.61 | 0.33 - 1.12 | 0.11 | 0.74 | 0.37 - 1.45 | 0.38 | 1.38 | 0.53 - 3.61 | 0.51 |
| **Past & now # University** | 1.16 | 0.68 - 1.97 | 0.58 | 1.02 | 0.60 - 1.73 | 0.94 | 1 | 0.55 - 1.80 | 0.99 | 0.62 | 0.23 - 1.69 | 0.35 |
| **Self-rated health ( reference: poor)** | | | | | | | | | | | | |
| **Never # Poor** | 1 | 1.00 - 1.00 | . | 1 | 1.00 - 1.00 | . | 1 | 1.00 - 1.00 | . | 1 | 1.00 - 1.00 | . |
| **Past only # Average** | 0.92 | 0.79 - 1.07 | 0.29 | 0.88 | 0.75 - 1.03 | 0.12 | 0.87 | 0.71 - 1.06 | 0.17 | 0.63 | 0.40 - 0.98 | **0.039** |
| **Past only # Good** | 0.81 | 0.60 - 1.09 | 0.16 | 0.88 | 0.65 - 1.18 | 0.38 | 0.72 | 0.52 - 1.02 | 0.062 | 0.67 | 0.38 - 1.18 | 0.16 |
| **Now only # Average** | 0.69 | 0.52 - 0.91 | **0.009** | 0.74 | 0.56 - 0.98 | **0.04** | 0.56 | 0.40 - 0.77 | **0.0005** | 0.6 | 0.31 - 1.16 | 0.13 |
| **Now only # Good** | 1.3 | 0.71 - 2.37 | 0.39 | 1.51 | 0.83 - 2.74 | 0.18 | 0.97 | 0.52 - 1.82 | 0.92 | 1.63 | 0.69 - 3.87 | 0.27 |
| **Past & now # Average** | 0.81 | 0.54 - 1.21 | 0.30 | 0.66 | 0.44 - 0.98 | **0.04** | 0.83 | 0.50 - 1.36 | 0.45 | 0.69 | 0.27 - 1.74 | 0.43 |
| **Past & now # Good** | 1.36 | 0.61 - 3.04 | 0.45 | 1.39 | 0.63 - 3.08 | 0.41 | 1.33 | 0.56 - 3.14 | 0.52 | 1.43 | 0.43 - 4.69 | 0.56 |

Multinomial regression. RRR: relative risk ratio. The base level of the outcome is: exposed to zero risk.

p-values <0,05 in bold.

Each stratification was estimated separately. Models were adjusted on sex, age, self-rated health, education, partnership status, presence of children, region, income category (stratification variable omitted as needed)
