# Supplementary material for: Association between unemployment and the co-occurrence and clustering of common risky health behaviors: Findings from the Constances cohort
Source: PLoS One. 2020 May 6;15(5):e0232262. doi: 10.1371/journal.pone.0232262 (PMC7202648; doi:10.1371/journal.pone.0232262)
Supplement: Attachment — Submitted filename: Response_Reviewers.docx [file pone.0232262.s003.docx]

Dear editor, dear reviewers

Here is our response to your reviews.

## Editor

2. We note that you have indicated that data from this study are available upon request. PLOS only allows data to be available upon request if there are legal or ethical restrictions on sharing data publicly. For information on unacceptable data access restrictions, please see <http://journals.plos.org/plosone/s/data-availability#loc-unacceptable-data-access-restrictions>.
In your revised cover letter, please address the following prompts:
a) If there are ethical or legal restrictions on sharing a de-identified data set, please explain them in detail (e.g., data contain potentially identifying or sensitive patient information) and who has imposed them (e.g., an ethics committee). Please also provide contact information for a data access committee, ethics committee, or other institutional body to which data requests may be sent.

*This is the data availability statement*

"Data Availability Statement: Personal health data underlying the findings of our study are not publicly available due to legal reasons related to data privacy protection. CONSTANCES has a data sharing policy but before data transfer a legal authorization has to be obtained from the CNIL (Commission nationale de l’informatique et des libertés), the French data privacy authority. The CONSTANCES email address is [contact@constances.fr."](mailto:contact@constances.fr.%22)

Additional Editor Comments:

The reviewers expressed several major concerns about the current manuscript. I would like to give the authors the chance to respond to these concerns and make relevant chances. However, please note that at this stage we cannot guarantee that your revised manuscript will be accepted for publication.

*We have thoroughly revised the manuscript, making good use of the reviewers’ advices, and hope to have raised the doubts expressed by reviewers and editor.*

In your revision note, please include EACH comment of the editor/reviewers, provide your reply, and when relevant, include the modified/new text (or motivate why you decided not to modify the text).

Reviewer #1 stressed the fact that the results section is particularly long and very difficult to follow. The use of multinomial regression instead of logistic regression should be considered. The authors were also asked to elaborate more on potential mechanisms, as this is not emphasized sufficiently in the discussion.

*We used multinomial regressions as suggested by Rev#1. We also explored interactions as Rev#1 also suggested. This led us to revise the methods, results and discussion. As a consequence we have deleted all the part that was very difficult to follow and focused on other aspects of the results.*

Reviewer #4 had made a comment about the novelty of the results. Please note that the PLOS ONE criteria for publication do not include an assessment of scientific novelty or innovation (<https://journals.plos.org/plosone/s/criteria-for-publication>). This comment will therefore not affect our final conclusion.

## Reviewer #1:

In this manuscript, results are reported of a cross-sectional study evaluating the association between unemployment and the co-occurrence and clustering of common risky health behaviors. The authors used data from more than 65.000 men and women from the French Constances cohort. They found that current unemployment, and to a lesser extent past unemployment, was associated with increased engagement in common risky behaviors. However, unhealthy behaviors did not cluster among men or women experiencing unemployment. Apart from the results, the manuscript is well written. Most of the results, however, are presented less clearly and succinctly.

*When taking into account the other comments, we have rewritten most of the results section. The parts that we have cut where the most obscure.*

Below are a few comments and questions for the authors’ consideration.
- The sample is restricted to complete data. The authors should provide information how many individuals are dropped because of missing data. The authors should also discuss potential problems due to missing at random (MAR) and not missing at random (NMAR). What is also unclear is that in the discussion it is stated that self-employed individuals and inactive populations (I would not define students and retirees as inactive) were excluded, but no mention of this was made in the methods section and it is unclear why these people were excluded.

*We agree that the exclusion of participants with missing data is a potential issue, but we believe that this issue is not really the representativeness of the cohort which is already not representative in the first place, given the very low participation rate to the cohort that necessarily leads to a healthy cohort as shown by supplemental table. The selection of individuals without missing data simply leads to an even healthier cohort as documented by the comparison of the characteristics of excluded and included volunteers (second supplementary table). In these conditions, the critical issue is the external validity of the study which is difficult to appreciate until similar analyses are performed in other cohorts with very different characteristics, and this issue is not created (only accentuated) by the exclusion of volunteers with missing data. Besides, the non-monotonous distribution of missing values in the cohort would require to use multivariate imputation by chained equations (MICE) which is time-consuming and not worth it given that this will not solve the issue of the external validity of the study. Moreover, as pointed out by Allison and more recently by White and Carlin, multiple imputation approaches are not devoid of potential bias.*

*Allison PD (2001) Missing data. Sage University Papers Series on Quantitative Applications in the Social Sciences 07–136.*

*White IR, Carlin JB (2010) Bias and efficiency of multiple imputation compared with complete-case analysis for missing covariate values. Stat Med 29: 2920–2931.*

*As regards self-employed workers or farmers: the sampling design includes workers as long as they are or have been salaried workers (wage earners). At the time of data collection in France, only salaried workers were covered by the unemployment insurance scheme. The experience of job loss was probably different for the self-employed.*

*We made it clearer in the methods (Study population and Statistical analysis) and discussion that our study covers people who are or have been salaried workers:*

[Introduction - Study population]The 15% excluded were mostly farmers or self-employed workers who had never worked as salaried workers

[Discussion] A first one is that, consistently with the French legislation regarding unemployment at the time of inclusion, the study selected only salaried or formerly salaried participants.

*We deleted the word “inactive”.*

Furthermore, what was done with disabled people or individuals who had no income?

*We excluded people who declared they were not working because they were not working for health reasons, as well as student and retirees : See Materials and Methods/Statistical analyses :*

Participants who declared they were not in employment because they were retired, studying, or for health reasons were excluded.

- Confounding variables are controlled for in the cross-sectional analyses. The authors should discuss why they only applied one model, rather than two or three. For instance, income is included in the model, but income is likely to be one of the mechanisms how unemployment affects health behaviour. To study whether this is the cause, it is plausible to include this variable in an additional model and then discuss whether results do or do not change when including this variable. Furthermore, it should be discussed that important confounding variables (e.g. education and BMI) are missing.

*Because we modified our modelling strategy and skipped the detailed analysis of risk combinations, we were able to present 3 models, adding up covariates (table 2):*

M1: adjusted on sex and age

M2: M1 + self-rated health, education, partnership status, presence of children, region

M3: M2 + income category

*Education is included in models 2 and 3. Self-rated health is also included, so BMI did not appear useful.*

- From the methods section it remains unclear what the exposure and outcome is. Please clarify and it would help as the exposure and outcome are described in a consistent order.

*We clarified this by making more explicit subsection titles in the Methods section:*

Exposure variable: employment status; outcome variables; covariates…

- Separate analyses were done for men and women, but no tests for interaction were made and reported. I would suggest to test this first, before reporting the results separately. Furthermore, I would recommend testing for interaction with income and age too (and eventually reporting the separated analyses if necessary).

*We applied this suggestion. We systematically tested interactions and report results. This allowed us to skip the separate analyses for men and women and emphasize other aspects of the results.*

*[Results]* When we examined interactions between covariates and experience of unemployment in the fully adjusted model, there were statistically significant interactions for age (chi-square test p = 0.015), self-rated health (p = 0.052) and education (p = 0.036). Association between risk co-occurrence and unemployment did not vary across gender, partnership status, or income category.

- The current health behavior variables are very crude. What is the reason for that no distinctions are made with non-smokers or ex-smokers (in the case of smoking) or with low, moderate or high physical activity (in the case of physical activity) and with low risk drinker, non-drinker, rarely drinker and risky drinker (in the case of alcohol consumption) and other variables? I recommend the authors to include more detailed exposure variables.

*When studying clustering, one needs dichotomous exposure variables (exposed or not) in order to compute the number of co-occurring risks. This is why our variables are not more detailed.*

- It is unclear to me why the authors used logistic regression analyses. Why not using a multinomial logistic regression? The multinomial model is an extension of the logistic regression model that allows for more than two categories in the response variable (i.e., number of risky health behaviours). Furthermore, what is the P for trend?

*We put this suggestion to use. multinomial models, resulting in Relative risk ratios rather than OR were used. They produce slightly more significant results. We also computed P for Trends. (Table 2)*

- The results section is very long and difficult to follow. Some sentences include more than 8 sentences and too many numbers and ORs are presented. Please restructure and only present the main findings. Furthermore, the odds ratios presented on page 10 are not included in a table.

*this section was entirely revised, focusing on number of co-occurring risks, interactions, clustering. the combinations of risks are treated very briefly with only one table (Table 3)*

From table 2 it appears that the O/E of risky health behavior is higher in those who have never been unemployed. I find that hard to understand, shouldn’t that be the other way around? Please explain.

*Our main explanation is that the unemployed have very high expected exposure rates, and the observed rates cannot rise higher than 100 %, so that the O/E ratio cannot rise as high as for those never unemployed. besides, the differences across unemployment status are not significant. We acknowledge this in the result section.*

- The mechanisms explained in the discussion are too general. Specific arguments are missing: arguments should be provided why unemployment is associated with smoking and unhealthy diet as the associations are not obvious, e.g. as unemployment may also provide more time for healthy cooking and makes smoking less affordable. Furthermore, please only concentrate on studies using similar study populations, rather than studies using data from the general population.

*We gave a little more details in the conclusion, in the last paragraph.*

- The cross-sectional design of this study is a huge limitation. The authors should discuss this as a limitation in their discussion. Another limitation that should be added is that, next to the risk factors, employment status is self-reported too.

*We did so :*

[Discussion] This supports the view that the increased prevalence and co-occurrence of risky behaviors would be a consequence of unemployment and that this might contribute to poor health in unemployed people [2, 3], **although analyses on prospective data are required to support this assumption.**

- Could the authors emphasize on the implications of this study and what are their suggestions for further research?

*This was included in the Discussion section:*

Such policies, however should be adapted to each health risk and would benefit from a practice-based approach, acknowledging that they constitute distinct social practices with specific meaning, context, and embeddedness in social relations [66]. In addition, more research is need regarding the co-occurring adoption of risky health behavior as people become unemployed, on prospective data.

## Reviewer #2:

The authors explored the co-occurrence and clustering of risky health behaviors in currently and/or formerly unemployed men and women. This manuscript is based on an impressive dataset and fills a gap of knowledge on clustering of these risky health behaviors in the unemployed, thus making a valuable contribution to the existing literature. However, there are issues that need to be addressed. In my opinion, the most important one is the brief description of the independent variables that quantify the risky health behaviors. The manuscript can be improved substantially when the authors provide more information on the questionnaires, describe the cleaning process, justify the cut-offs and if necessary, repeat the analyses.

*We gave more details and references regarding how the risky behaviors were measured (Materials and Methods/Outcome variables)*

Introduction : Many if the examples that the authors provide in the Introduction are based on studies in American populations. As the social programs, social security systems and health care systems of the limited welfare state in the US is significantly different compared to these systems in Western European countries, please focus your examples on the latter.
In addition, please include one or two lines regarding the differences in men and women as these separate analyses are discussed in the Methods, Results and Discussion sections but not in the Introduction.

*The lack of evidence from France and more generally Europe is now acknowledged in the introduction. In addition, thanks to Rev#1 suggestions, we substantially modified our models, revised the results and skipped the gender comparison.*

To our knowledge, however, no study has examined to which extent common behavioral risk factors cluster in unemployed people.

Page 7 line 155-165
Is ‘at baseline’ the same as ‘at inclusion’? I.e. were all of these measurements done at the same time in one of the 21 medical centers? If so, please consistent terminology throughout the manuscript.

*We replaced occurrences of “at baseline” by “at inclusion”.*

Page 7 line 155-165
Please provide some details on the questionnaires used or refer to a published manuscript on these questionnaires, especially on food frequency and physical (in-)activity. The marker for an unhealthy diet – consuming fruits or vegetables less than three times per day – might be too unspecific. How does this, for instance, relate to the recommendations by WHO? (>400 grams of fruits and vegetables per day).

*the 3/day cutoff for fruit and vegetable is the definion of “low FV consumer” in French nutrition policy. we specified that.*

[Methods]. People were considered at risk if the sum of their fruit (fresh or squeezed) and vegetable (raw or cooked) consumption was lower than three times per day everyday (not just the day before the questionnaire). This cut-off is consistent with definitions of low fruit and vegetable consumption in French health policy [47].]

The authors define physical inactivity as less than 30 minutes of physical activity, defined by walking or cycling, exercising, gardening and housekeeping, over a regular week. Almost 9 out of 10 participants met this requirement (Table 1), which is comforting, but the differences in physical (in-)activity in leisure time might increase after those 30 minutes. The recommendations for adults is +/- 30 minutes of moderate physical activity per day, for at least 5 days a week, including walking, cycling, housework, gardening etc. Please clarify and justify the chosen cut-off of 30min/week.

*We gae extra detail on data collection including previous publications. also we modified the cutoff point for physical inactivity – now low physical activity and gave further references regarding the measurement. See methods – outcome variables.*

[Methods] Leisure-time physical activity was determined by a calculated score ranging from 0 (i.e. being very active) to 6 (being not active at all). The physical activity questionnaire asked about regular practice of walking or cycling; practicing a sport; gardening or housekeeping over the past 12 months. Each of the three items was noted 0 if the answer was no; 1 if practice was regular but low (less than 15 minutes for sports or 2 hours for the other two, weekly); 2 if practice was regular and higher. Data regarding the score obtained by summing the three items has been published [48]. People with a score below three were considered at risk.

What is the definition of ‘smoker’ in this study? How was this assessed (how many cigarettes per day/week)? Were consumers of e-cigarettes or cannabis-users removed from the analysis/set to missing/..?

*E-cigs and cannabis are not considered as smoking in this study. this is stated in the text:*

Smoking was understood as participants who smoked at inclusion and did not comprise the consumption of e-cigarettes or cannabis.

Page 10 line 122-216
Please rephrase this sentence for clarification. If I read correctly, ‘their exposure’ and ‘they’ refers to participants who were unemployed at baseline with or without past experience of unemployment, but to make this more clear please indicate which group is described. In addition, please clarify the last part of the sentence ‘alcohol abuse becoming more frequent than leisure-time physical activity’.

*Done.*

Page 11 line 237
‘…one, two or three factors significantly lower’ There is a word (‘were’?) missing from this sentence. Also, depending on your chosen significance level (which was not stated in the Methods section) a p value of .03 or .08 in the fully adjusted model is or is not significant.


Page 14 line 300-302
‘Prevalence rates of exposure to non or only one risk factor were at the higher end while rates of exposure to two, three or four factors were at the lower end of the distributions described in other Western populations.’ On page 11 line 239-243 the authors state that that the exposure to a single factor was almost the most frequent, followed by those to two, none, three and finally four. Please clarify this difference.

*Done:*

Prevalence rates of exposure to none or only one risk factor were at the higher of the distributions described in other Western populations end while rates of exposure to two, three or four factors were at the lower end [47–55].

Page 15 line 322-327
The reasons for the low fruit and vegetable intake among all participants and low prevalence of leisure-time physical inactivity might lie in the instruments or cut-offs the authors used to create their dichotomous variables. See also my comments for Page 7 line 155-165.

*We put this advice in practice and chose less stringent cutoff values. It improved the quality and statical significance of our results. See methods and results sections, and talbe 1 for prevalences*

*We also addressed the minor comments.*

## Reviewer #3:

Line 116-118: The distinction between co-occurrence and clustering is basic. This distinction should be explained more clearly and elaborated a little in order to facilitate for the reader.

*Done :*

[Introduction] : Co-occurrence, i.e., the number of risks factors participants were exposed to, can be distinguished from clustering, understood as the co-occurrence of risk factors with greater frequencies than expected by chance [43].]

[Methods] Clustering of risk factors refers to the co-occurrences of risk factors with greater frequencies than expected by chance, i.e. if exposure to each risk were independent of one other [43]. For each number of co-occurring risk factors, expected rates of co-occurrence were computed from the prevalence of each risk factor assuming that they occurred independently. Clustering was defined as observed to expected prevalence ratios significantly >1. We also computed the frequency and clustering of each specific risk combinations.

Line 296: There is no discussion of how the high drop-out rate (92.7 per cent as I can figure out) may affect the results. Could the low unemployment rate at the inclusion be due to this?

*We corrected this. We also provide a supplementary table comparing the population included in the study with a random sample of French with similar age and employment status.*

[Discussion] A first one is that the study selected only salaried or formerly salaried participants, excluding self-employed or farmers (who were not covered by the French unemployment insurance system at the time of the study). Second, the very low participation rate resulted in the selection of motivated and socially privileged individuals even though the stratified sampling strategy tried to compensate for the higher non-response of low socioeconomic status persons. This is illustrated by the comparison of cohort participants with randomly selected workers [63] in the same age range. The proportion of participants with university education is very high and the unemployment rate is somewhat lower (Supplemental table).

All the minor points were addressed when revising the manuscript

## Reviewer #4:

• The article investigates the association between unemployment and the co-occurrence of risky health behaviors (clustering). It uses data form the Constances cohort.

• The article hardly presents anything new. I guess we all know that there’s a higher prevalence or occurrence and even co-occurrence of risky behavior in unemployed populations. I sort of miss a good and convincing story here.

*The journal editor advised us that novelty is not a criterion for publication in PLosOne. However, we highlighted that clustering of risky behavior has not been measured before, especially in France.*

[Introduction ] To our knowledge, however, no study has examined to which extent common behavioral risk factors cluster in unemployed people.

• It would for example be interesting to investigate the association between the clustering of risky behavior and some health outcomes. What is the interaction effect between the different kinds of unhealthy behavior? For instance, is the impact equal to the sum of the impact of smoking + the impact of drinking or is there an interaction effect, the one strengthening the impact of the other (by gender and age group for example). I have the feeling there was a lot more potentiality in the data…

*We clarified the difference between risk co-occurrence (which is, indeed, expected) and risk clustering, i.e. the co-occurrence of risks with a probability higher than expected, had each risk be statistically independent. This has never been measured for the unemployed before.*

*Our results show that there is higher risk co-occurrence but no clustering. In other words the risk co-occurrence is not higher than expected given the prevalence rate of each risk factor among the unemployed and the employed.*

*As regards health outcomes of clustered risks, it is a very important issue but it would exceed the scope of the current paper. It could be the next step of our research.*

• The authors do not present a clear theoretic framework in which the mechanisms are discussed.

*We developed the practice theory approach in the last paragraph of the discussion.*

[Discussion] This might be due to the fact that engagement in risky behaviors relies on mechanisms that differ from one behavior to another, hence, the prevalence of one behavior can increase independently of the others. Engagement in risky behaviors may be related to strategies for coping with stress but may also involve the multiple understandings of when and how much it is appropriate to consume alcoholic drinks, cigarettes or healthy foods, or engage in any practice that generates physical activity [64]. This suggests that preventive strategies addressing risky behaviors need not target the unemployed specifically, because they are overexposed but according to similar patterns of co-occurrence. Preventive policies however, should be adapted to each health risk and would benefit from a practice-based approach, acknowledging that they constitute distinct social practices with specific meaning, context, and embeddedness in social relations [65]. In addition, more research is need regarding the co-occurring adoption of risky health behavior as people become unemployed, on prospective data.

• Selection effects are nowhere mentioned, while it is clear in literature that these are an important dimension explaining part of the association.

*We have addressed this point in the Discussion :*

A first one is that, the study selected only salaried or formerly salaried participants, excluding self-employed or farmers (who were not covered by the French unemployment benefit system at the time of the study). Second, the very low participation rate resulted in the selection of motivated and socially privileged individuals even though the stratified sampling strategy tried to compensate for the higher non-response of low socioeconomic status persons, as illustrated by the comparison of cohort participants with randomly selected workers [63] in the same age range. The proportion of participants with university education is very high and the unemployment rate is somewhat lower (Supplemental table). Third, unemployment status and behavioral risk factors were self-reported.

• The style of writing still could improve considerably.

*We improved the style of writing by revising the results section. We got rid of the tedious description of risk combinations and polished the style throughout.*
