# Supplementary material for: Association between unemployment and the co-occurrence and clustering of common risky health behaviors: Findings from the Constances cohort
Source: PLoS One. 2020 May 6;15(5):e0232262. doi: 10.1371/journal.pone.0232262 (PMC7202648; doi:10.1371/journal.pone.0232262)
Supplement: Attachment — Submitted filename: Comments_Paper Plos One_2019-R1.docx [file pone.0232262.s004.docx]

Thank you for answers. Although the manuscript has improved significantly, I still have a few issues to consider. What remains unclear to me is how many people were excluded from your study population. It would help if you could provide a flow diagram that shows the number of included and excluded participants. How many participants were eligible and how many were further excluded? From the current methods section it is not clear what number of participants you start with. Several inclusion criteria are then mentioned, but actual numbers of participants that were excluded are not given, apart from the final number which is 66,630. Besides, information on inclusion criteria are mentioned in both paragraphs of the ‘study population’ (regarding informed consent, etc) and the ‘statistical analysis’ (regarding e.g. retired participants), which is confusing. And what do you mean with that a complete dataset was available? How many participants did not have complete data and were therefore excluded? This is essential information to put your findings into context. A comparison of several characteristics of the in- and excluded would help too.

Furthermore, the logic behind your statement that ‘self-rated health is included into the models, so BMI did not appear useful’ is very difficult to follow. In my opinion BMI and self-rated health are completely different variables, which could potentially be included both into the models. Please elaborate.

Also, although the interaction with age, self-rated health and education is statistically significant, stratified analyses for all three variables were not presented. It is therefore not clear what direction the interaction goes to and how these results should be interpreted. Please provide stratified analyses and revise your results and discussion accordingly.

Results section: what does a most frequent combination of risky behaviors of ‘no risk’ mean? Again, this section is particularly hard to follow. Please revise.
