# Supplementary material for: Association between unemployment and the co-occurrence and clustering of common risky health behaviors: Findings from the Constances cohort
Source: PLoS One. 2020 May 6;15(5):e0232262. doi: 10.1371/journal.pone.0232262 (PMC7202648; doi:10.1371/journal.pone.0232262)
Supplement: Attachment — Submitted filename: Rebuttal R2.docx [file pone.0232262.s005.docx]

PONE-D-19-26293R1

## Additional Editor Comments (if provided):

Both reviewers appreciated the revisions made by the authors, but highlighted some remaining issues, that can be addressed in a final, minor revision round.

*Thank you. We will keep this in mind when addressing reviewers’ comments.*

## Review Comments to the Author

### Reviewer #1:

Although the manuscript is improved, I still have a few issues to consider. I think the authors are getting off easy sometimes and a few of my questions were not sufficiently answered. One example is that stratified analyses are not presented even though statistical significant interactions with three covariates were found. It might be the results are even stronger in low educated people, but estimates and further explanations are not given, which makes it very hard to interpret these results.

*Please see below.*

### Comments in attached file

*As this was not clearly indicated, we have assumed that this file contained the longer version of Reviewer #1’s review.*

Thank you for answers. Although the manuscript has improved significantly, I still have a few issues to consider. What remains unclear to me is how many people were excluded from your study population. It would help if you could provide a flow diagram that shows the number of included and excluded participants. How many participants were eligible and how many were further excluded? From the current methods section it is not clear what number of participants you start with. Several inclusion criteria are then mentioned, but actual numbers of participants that were excluded are not given, apart from the final number which is 66,630. Besides, information on inclusion criteria are mentioned in both paragraphs of the ‘study population’ (regarding informed consent, etc) and the ‘statistical analysis’ (regarding e.g. retired participants), which is confusing. And what do you mean with that a complete dataset was available? How many participants did not have complete data and were therefore excluded? This is essential information to put your findings into context. A comparison of several characteristics of the in- and excluded would help too.

*As suggested, we grouped all the information on the sample under the section “study population”. We introduced information on the number of eligible participants and those actually included in the study population.*

In the cohort at the time of data extraction there were 91,259 adults aged 18 to 65, currently or previously salaried workers, who declared that they were currently either employed or unemployed and for whom lifestyle, occupational exposure and professional schedule questionnaires were completed. We excluded individuals who declared they were not working for health reasons (149 unemployed) and who had missing data on covariates (12,172) or dependent variables (13,308). This resulted in a study population of 65,630 participants. Among them, 51,875 (79.1%) were never unemployed, 9182 (14%) were unemployed in the past but not at inclusion, 3160 (4.8%) were unemployed at inclusion but not in the past and 1413 (2.1%) were unemployed both in the past and at inclusion.

*We did not compare the incomplete and complete cases. Such comparison would be of interest but would neglect those who did not participate at all in the study. Instead we provided a comparison between our study population and a nationally representative survey of workers (supplementary table S1 which is reproduced below):*

Baseline sociodemographic characteristics of CONSTANCES participants compared to randomly selected workers.

|  | **CONSTANCES**  **participants** | **Randomly**  **selected individuals** | **p** |
| --- | --- | --- | --- |
| **N** | 65,630 | 14,597 | - |
| **Age, y (mean ± SD)** | 42.7 ± 10.4 | 39.9 ± 10.7 | <0.0001 |
| **Men,** **% (n)** | 48.6 (31,915) | 50.9 (7436) | <0.0001 |
| **Unemployment rate, % (n)** | 7.0 (4573) | 10.4 (1524) | <0.0001 |
| **Education, % (n)** |  |  |  |
| Primary, lower secondary | 17.6 (11,558) | 52.2 (7626) |  |
| High school diploma | 15.3 (10,034) | 16.2 (2363) | <0.0001 |
| University | 67.1 (44,038) | 31.6 (4608) |  |

Univariate comparisons were performed with chi-square test (or Fisher’s exact test when necessary) for categorical variables and with Student’s t-test for continuous variables. Randomly selected workers in the same age range (18–65 years) than CONSTANCES participants at baseline are from the decennial health survey conducted in 2003 by the French National Institute of Statistics and Economic Studies.

Furthermore, the logic behind your statement that ‘self-rated health is included into the models, so BMI did not appear useful’ is very difficult to follow. In my opinion BMI and self-rated health are completely different variables, which could potentially be included both into the models. Please elaborate.

*We do not deny that BMI is a different construct, nor that it may be of interest in the study of the relationship between unemployment and behavioral risk factors. However in our model, self-rated health is only a control variable, and BMI would have the same status,* *were it introduced. When selecting covariates, we had to take into account the fact that many variables (including health, income, and BMI) might be both causally anterior to unemployment, and causally posterior to exposure to risk factors. This means that there was a risk of over-controlling for confounding variables. For this reason, we added covariates to the models progressively (model 1 to 3), and we were reluctant to add any additional covariate to our model. Since there is a (inverted U-shaped) relationship between BMI and self-rated health, we decided against adding BMI on top of self-rated health, which captures, we assume, more dimensions of health status.*

Also, although the interaction with age, self-rated health and education is statistically significant, stratified analyses for all three variables were not presented. It is therefore not clear what direction the interaction goes to and how these results should be interpreted. Please provide stratified analyses and revise your results and discussion accordingly.

*We had not presented the results in a table because the table is large and shows only little significant results. However, you are right to point that this is a result too. We have added the details of the stratified results in supplementary table 2 (see an image below) and added the following sentences of comment in the text:*

When we examined interactions between covariates and experience of unemployment in the fully adjusted model, there were statistically significant interactions for age (chi-square test p = 0.015), self-rated health (p = 0.052) and education (p = 0.036). **However, when we stratified models over these variables, there was no pattern or trend in the relative risk ratios (Supplementary table S2).** Association between risk co-occurrence and unemployment did not vary across gender, partnership status, or income category.

*And in the discussion section :*

**Stratified analyses indicated that** among unemployed participants, there was no variation in risk co-occurrence across gender, household status or income category.

Results section: what does a most frequent combination of risky behaviors of ‘no risk’ mean? Again, this section is particularly hard to follow. Please revise.

*This was confusing, we erased the mention of “no risk”. We also rephrased slightly the paragraph. Also we used an abbreviation for “fruit and vegetable” in all the paper in order to ease reading.*

As shown in Tables 3 and 4, the three most frequent combinations were low FV intake (30.9 to 40.3%), low FV intake & low physical activity (13.7 to 18.7%), and low FV intake & smoking (9.3 to 16.1%), with slight variations in the frequency order across experience of unemployment. The two combinations with the highest observed-to-expected ratios were the four risks (O/E 1.95 to 2.70), and FV & smoking & alcohol abuse (1.63 to 1.92). Beyond that, the order of risk combinations varied across experience of unemployment.

### Reviewer #2:

The authors made significant changes to the manuscript, which improved the quality of the article substantially. However, the language used might need some improving/polishing.

*We have revised the English. We have introduced an abbreviation for fruit and vegetable (FV) which, we hope, improves readability.*

Other comments:

- This manuscript explores the relationship of risky health behaviors with the (un)employment. The added value of this manuscript is the examination of the extent to which common behavioral factors cluster in unemployed individuals. The introduction provides us with many examples of the association between these risk factors and unemployment, but this part of the manuscript could benefit by focusing on the definitions of co-occurrence and clustering of risky health behaviors (in the very beginning) and the implications for social science or health policies (instead of simply demonstrating the association between health factors and (un)employment).

*As suggested, we have revised the introduction: we have* *introduced a paragraph* *on health consequences of the co-occurrence and clustering of risky behaviors and a statement on the potential implication of the study for the* *design of preventive strategies among unemployed people .*

The co-occurrence of behavioral risk factors dramatically increases the effects on health and mortality. Thus, old European men and women who combine adherence to a Mediterranean diet, moderate alcohol use, being physically active and non-smoking have a mortality rate one third of that which is observed in those who do not adopt any of these behaviors {Knoops et al., 2004, #36616}. Similarly, middle-aged and older UK men and women who combine non-smoking, being physically active, moderate alcohol intake and fruit and vegetable intake of at least five servings a day have a 4-fold difference in the risk of dying over an average period of 11 years compared to those who do not adhere to any of these behaviors {Khaw et al., 2008, #5434}. In addition to the mere co-occurrence of behavioral risk factors, there can exist a clustering of these factors, i.e., a higher frequency of co-occurrence than expected on the basis of the prevalence of each factor, that can exacerbate the effects on health and mortality [43]. To our knowledge, despite the large body of evidence showing that unemployment is associated with a high exposure to common behavioral risk factors, no study has examined the co-occurrence and clustering of these factors among unemployed people. One report has

- Line 171-177 “Leisure-time physical activity was determined by a calculated score ranging from 0 (i.e. being very active) to 6 (being not active at all).” According to the explanation of the scoring in the following sentences, I assume a score of 0 is associated with being not active at all and 6 with being very active. However, as in Table 1 ‘No’ is mainly associated with a favorable outcome, it could be that this variable recoded?

*In the questionnaire, 0 is inactive, but in the table we code the* risk of low *physical* *inactivity, so a “no” is indeed a healthier situation. We clarified this in the text.*

Data regarding the score obtained by summing the three items has been published [48]. People with a score below three were considered at risk of low physical inactivity.

- I understand the aim of the authors to explore clustering of risky behaviors, for which dichotomous variables are needed. This required crude (and sometimes arbitrary) cut-offs for the risk factor-variables and those might be of consequence for your conclusions, especially those on the implications of the study.

*We added a sentence in the paragraph that dealt with limitations.*

Last, the cutoff points for exposure to risks, although chosen consistently with French public health guidelines, might affect the results.

- Line 236-240 Please rephrase, as this sentence is quite long and I’m not sure which closing paired bracket belongs to which opening paired bracket.

*We rephrased as follows:*

Unemployed participants reported more often low FV intake. The minimally adjusted OR (95% CI) was 1.38 (1.25-1.53) for the unemployed at inclusion without past experience of unemployment. The unemployed were also more exposed to smoking (2.06 (1.91-2.23)) and alcohol abuse (1.78 (1.60-1.96)), but not low leisure-time physical inactivity (1.02 (0.94-1.10), p = 0.60, and 1.10 (0.97-1.23-1.31), p = 0.11). For the unemployed with past experience of unemployment, the OR were slightly but not significantly larger than for those without past experience.

- The authors report that unhealthy behaviors did not cluster more among participants experiencing unemployment than among those who are employed. They suggest that “preventive strategies addressing risky behaviors need not target the unemployed specifically, because they are overexposed but according to similar patterns of co-occurrence.” Line 345-366. Besides the fact that this sentence might need some polishing, I’m not sure whether I understand the main message of this sentence.

*Indeed. We deleted this awkward sentence and revised the last paragraph*

This might be due to the fact that engagement in risky behaviors relies on mechanisms that differ from one behavior to another, hence, the prevalence of one behavior can increase independently of the others, **whatever the employment status.** Engagement in risky behaviors may be related to strategies for coping with stress but may also involve multiple understandings of when and how much it is appropriate to consume alcoholic drinks, cigarettes or healthy foods, or engage in any practice that generates physical activity [64]. This suggests that preventive strategies should be **adapted to each unhealthy behavior in unemployed as in employed people** and would benefit from a practice-based approach, acknowledging that they constitute distinct social practices with specific meaning, context, and embeddedness in social relations [65].

- Please provide chapter or page numbers for reference [65].

*Done*

Cohn S. From health behaviours to health practices: an introduction. Sociology of Health & Illness. 2014;36: 157–162. doi:[10.1111/1467-9566.12140](https://doi.org/10.1111/1467-9566.12140)

7. PLOS authors have the option to publish the peer review history of their article ([what does this mean?](https://journals.plos.org/plosone/s/editorial-and-peer-review-process#loc-peer-review-history)). If published, this will include your full peer review and any attached files.

If you choose “no”, your identity will remain anonymous but your review may still be made public.

**Do you want your identity to be public for this peer review?** For information about this choice, including consent withdrawal, please see our [Privacy Policy](https://www.plos.org/privacy-policy).

Reviewer #1: Yes: Gerrie-Cor Herber

Reviewer #2: No

[NOTE: If reviewer comments were submitted as an attachment file, they will be attached to this email and accessible via the submission site. Please log into your account, locate the manuscript record, and check for the action link "View Attachments". If this link does not appear, there are no attachment files to be viewed.]

While revising your submission, please upload your figure files to the Preflight Analysis and Conversion Engine (PACE) digital diagnostic tool, <https://pacev2.apexcovantage.com/>. PACE helps ensure that figures meet PLOS requirements. To use PACE, you must first register as a user. Registration is free. Then, login and navigate to the UPLOAD tab, where you will find detailed instructions on how to use the tool. If you encounter any issues or have any questions when using PACE, please email us at [figures@plos.org](mailto:figures@plos.org). Please note that Supporting Information files do not need this step.

*In compliance with data protection regulations, you may request that we remove your personal registration details at any time.* [*(Remove my information/details)*](https://www.editorialmanager.com/pone/login.asp?a=r)*. Please contact the publication office if you have any questions.*
